# Associations between cancer diagnosis and patients’ responses to an inpatient tobacco treatment intervention

**DOI:** 10.1002/cam4.4082

**Published:** 2021-07-01

**Authors:** Amanda M. Palmer, Alana M. Rojewski, Georges J. Nahhas, K. Michael Cummings, Graham W. Warren, Benjamin A. Toll

**Affiliations:** ^1^ Department of Public Health Sciences Medical University of South Carolina Charleston SC USA; ^2^ Department of Pulmonary, Critical Care, Allergy, and Sleep Medicine Medical University of South Carolina Charleston SC USA; ^3^ Hollings Cancer Center Medical University of South Carolina Charleston SC USA; ^4^ Department of Psychiatry and Behavioral Sciences Medical University of South Carolina Charleston SC USA

**Keywords:** cancer, cessation, hospitalization, inpatient, prevention, smoking, tobacco

## Abstract

**Background:**

Diagnosis of a chronic illness, such as cancer may influence health behavior changes, such as smoking cessation. The present analyses examine associations between a cancer diagnosis (i.e., yes or no) and response to an opt‐out smoking cessation bedside intervention provided to hospitalized patients. It was hypothesized that patients with a past or present cancer diagnosis would report higher motivation and engagement with quitting smoking, and higher rates of smoking abstinence after hospital discharge, compared to those without a cancer diagnosis.

**Methods:**

Chart review was conducted on 5287 inpatients who accepted bedside treatment from a counselor and opted‐in to automated follow‐up calls from July 2014 to December 2019.

**Results:**

At the time of inpatient assessment, those with a past or present cancer diagnosis (*n* = 419, 7.9%) endorsed significantly higher levels of importance of quitting than those without a cancer diagnosis (3.92/5 vs. 3.77/5), and were more likely to receive smoking cessation medication upon discharge (17.9% vs. 13.3%). Follow‐up data from 30‐days post‐discharge showed those with a cancer diagnosis endorsed higher rates of self‐reported abstinence (20.5%) than those without a cancer diagnosis (10.3%; *p* < 0.001).

**Conclusion:**

Being hospitalized for any reason provides an opportunity for smokers to consider quitting. Having a previous diagnosis of cancer appears to increase intention to quit and lead to higher rates of smoking cessation in patients who are hospitalized compared to patients without cancer. Future research needs to work toward optimizing motivation for smoking cessation while admitted to a hospital and on improving quit rates for all admitted patients, regardless of diagnosis.

## Graphical abstract

This study examined responses to an inpatient tobacco treatment program between cancer and non‐cancer patients admitted to a large hospital. Results showed that during the time of hospitalization, patients with cancer felt that quitting smoking was more important and were more likely to be given medications to quit at discharge. One month after discharge, patients with cancer were more likely to have quit.

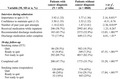

## 1 INTRODUCTION

Smoking by cancer patients and cancer survivors is causally linked to several adverse health outcomes.^1^ Fortunately, quitting smoking even after a cancer diagnosis improves health and survival.^2^, ^3^ Smoking cessation benefits cancer patients by improving cancer‐related outcomes (e.g., reduces the risk of secondary tumors) as well as decreasing the likelihood of other smoking‐related health issues (such as heart disease, stroke, and pulmonary disease).^4^ For this reason, clinical practice guidelines from multiple oncology organizations emphasize the importance of providing smoking cessation treatments to all patients with cancer.^3^, ^5^

Given the health benefits of quitting smoking, hospitals and medical systems are encouraged to implement tobacco treatment programs into their care system.^6^, ^7^ It is recommended that tobacco treatment programs include specialized tobacco treatment clinicians that provide cessation counseling, resources, medications, or referral to external resources such as state quitlines.^8^, ^9^, ^10^ Despite systemic barriers to implementation, many established tobacco treatment programs have succeeded in efficiently integrating evidence‐based treatments into clinical settings.^10^, ^11^ Both providers and patients benefit from these services, as they reduce smoking‐related burdens on health, improve treatment efficacy, and reduce costs for the hospital.^12^, ^13^, ^14^, ^15^

Research^16^ suggests that some medical diagnoses (e.g., cancer) may serve as the foundation for increased motivation for health behavior change, such as smoking cessation. However, patients face barriers to quitting, including stigmatization associated with smoking, lack of cessation assistance, lack of institutional resources, lack of training, lack of time, and lack of prioritization of smoking cessation in the context of cancer care.^3^, ^17^, ^18^, ^19^

To overcome barriers related to access to evidence‐based care, opt‐out approaches have been utilized. In an opt‐out program, patients are identified through structured tobacco use screening and are automatically referred for smoking cessation support.^13^ After referral, the patient chooses their level of involvement in treatment. Not only are these interventions efficacious, but they have been proven to reduce readmission rates and costs.^20^, ^21^ In the outpatient setting, opt‐out approaches have been well‐received by cancer patients.^22^ However, little is known about the degree of effectiveness of these interventions among cancer patients, relative to those without such a diagnosis. The purpose of the present study is to examine outcomes from an opt‐out smoking cessation bedside consult intervention and the effect of a cancer diagnosis on outcomes and other therapeutic factors (e.g., importance of quitting).

## 2 METHODS

### 2.1 Setting and participants

The Tobacco Treatment Program (TTP) at the Medical University of South Carolina (MUSC) serves three inpatient hospitals within the MUSC system. Overall, the TTP is an integrated, comprehensive, opt‐out tobacco treatment program with both inpatient and outpatient services. The program is staffed by psychologists, pharmacists, and certified tobacco treatment specialists. The TTP receives daily notifications of all hospital inpatients, who report any current tobacco use at the time of admission.^21^ These patients are then visited by a clinician who conducts a brief interview and documents the encounter in the electronic health record at the bedside using an iPad. Patients who receive the bedside consult are also enrolled to receive automated, interactive voice recognition (IVR) calls 3‐, 14‐, and 30‐days post‐discharge to assess their smoking status and refer them to the South Carolina Quitline or outpatient counseling if desired. Patients may decline the bedside consult and/or the telephone follow‐up calls. Details of the patient tracking and follow‐up system used at MUSC can be found elsewhere.^20^, ^21^

The present analysis consists of data from participants who self‐reported tobacco use upon admission and completed an inpatient counseling session during their hospitalization between July 2014 and December 2019. Medical record numbers were used to link patients to the National Cancer Registry Database, and patients were coded as having a past or current cancer diagnosis if their date of cancer diagnosis occurred prior to or on the day of the inpatient admission. Follow‐up data were collected through the chart review of patients’ responses to the IVR telephone system 30‐days following discharge. This study was exempt from participant consent and approved by the MUSC Institutional Review Board.

### 2.2 Measures

#### 2.2.1 Demographics and smoking characteristics

Patients’ race and biological sex were collected from medical records. TTP clinicians asked patients which tobacco products they used over the past month (cigarettes, cigars, hookah, bidis, oral tobacco, or e‐cigarettes), how often they used the product(s) over the past month (daily or non‐daily), how many units were used per day (e.g., cigarettes smoked per day), and how soon after waking they used their first product (a proxy for dependence^23^).

#### 2.2.2 Importance to quit

The importance to quit was measured by asking “How important is quitting smoking to you on a scale of 1–5, with 5 being the most important?”

#### 2.2.3 Confidence in quitting

The importance to quit was measured by asking “How confident are you that you will be able to remain smoke free on a scale of 1–5, with 5 being the most confident?”

#### 2.2.4 Quit attempts in the past year

Patients were asked how many times, if any, they tried to quit smoking during the past year.

#### 2.2.5 Medication during hospitalization

Patients were asked if they had received a quit smoking medication (such as nicotine replacement therapy [NRT]) during hospitalization.

#### 2.2.6 Medication recommendation for discharge

Counselors discussed discharge medication options with patients, which included NRT, bupropion (Zyban), or varenicline (Chantix). A shared decision was made between the patient and counselor to recommend medications for discharge.

#### 2.2.7 Discharge medication

Counselors pended recommendations for medications to the attending physician in the medical record system. Chart review was used to determine if medication was approved and provided by the physician upon discharge.

#### 2.2.8 Seven‐day point prevalence abstinence

This was collected during the 30‐day IVR phone follow‐up, if completed. Patients were coded “quit” if they endorsed not smoking for the 7 days prior to the phone call. If not quit, participants were asked about the level of interest in quitting in the near future by endorsing “ready” or “not ready” to quit.

### 2.3 Analyses

Descriptive statistics were used to describe sample characteristics, including patient demographic information. Chi‐squared analyses and *t*‐tests were used to compare interview and follow‐up responses between cancer and non‐cancer groups. Follow‐up analyses were completed with both the sample of completed follow‐up responders as well as the full sample using an intent‐to‐treat (ITT) approach,^24^ coding non‐responders as smoking, as a sensitivity analysis. Research data originated from electronic health records and are not shared due to privacy restrictions.

## 3 RESULTS

### 3.1 Sample characteristics

**FIGURE 1:**
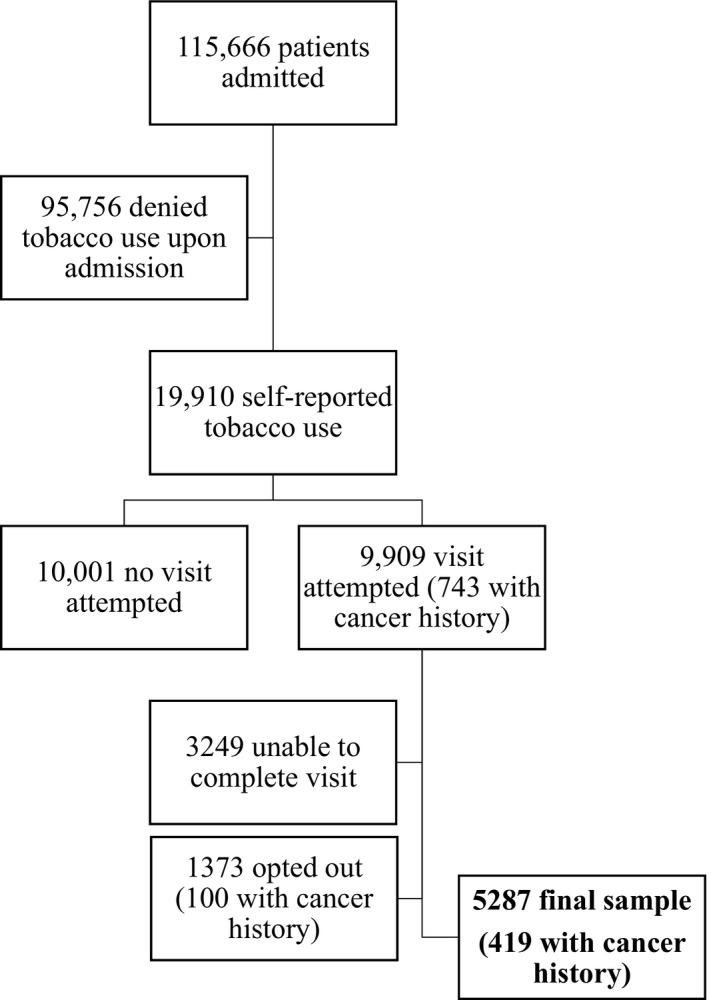
Flow chart of patient records reviewed. Visits were not attempted due to limitations of staff availability and/or patients discharged prior to visit attempt. Visits were unable to be completed due to patients being unavailable during the visit attempt.

A flow chart of patient records reviewed is shown in Figure 1. Of the 115,666 admitted patient medical records reviewed, 19,910 (17.2%) were self‐reported tobacco use upon admission. Of those, 9909 (49.8%) identified smokers were attempted to be visited by the TTP counselor, with 743 matched to a cancer diagnosis prior to admission using the National Cancer Center Registry database. Of all patients visited by the TTP, 100 (13.4%) patients with cancer diagnoses prior to admission and 1273 (13.9%) of patients without cancer opted out of the service. The remaining patients were unable to be counseled for a variety of reasons (e.g., not in the room, unresponsive).

Participant demographics and smoking information from the final sample (*N* = 5287) that completed the bedside interview and counseling are shown in Table 1. Of those, 419 (7.9%) were identified as having a cancer diagnosis prior to or during admission (leaving 4868 [92.1%] without). The most common cancer sites among patients identified included lung (*n* = 90), head/neck (*n* = 56), blood/bone (*n* = 44), brain (*n* = 32), and colorectal (*n* = 27). Generally, patients were middle‐aged and represented the racial and ethnic demographics of South Carolina. A large majority (91.8%) were cigarette smokers, with nearly 75% of patients endorsing daily smoking/other product use, 43.2% endorsing high dependence, and reporting smoking between half to one pack of cigarettes per day, on average. Patients with a history of cancer were slightly older, but there were no significant differences between patients with and without cancer diagnoses on all other demographic or tobacco use variables. Results of age‐adjusted analyses can be seen in the Table S1.

**TABLE 1:** Participant characteristics

Variable (*M* or *N*, SD or %)	Total sample (*N* = 5287)	Patients with cancer diagnosis (*N* = 419)	Patients without cancer diagnosis (*n* = 4868)	*p*
Age	45.32 (25.09)	48.95 (26.09)	45 (24.97)	0.002
Race/Ethnicity				
White	2819 (53.3%)	225 (53.7%)	2594 (53.3%)	0.525
Black/African American	1635 (30.9%)	117 (28.9%)	1518 (31.2%)	
Hispanic	67 (1.3%)	4 (<1%)	63 (1.3%)	
Asian	16 (<1%)	1 (<1%)	15 (<1%)	
Native American/Alaskan Native	22 (<1%)	4 (<1%)	18 (<1%)	
Other/Mixed	18 (<1%)	1 (<1%)	17 (<1%)	
Biological sex				
Male	2950 (55.8%)	247 (58.9%)	2703 (55.5%)	0.177
Female	2336 (44.2%)	172 (41.1%)	2164 (44.4%)	
Tobacco product use in past 30 days				
Cigarettes	4854 (91.8%)	390 (93.1%)	4464 (91.7%)	0.699
Cigars	299 (5.7%)	17 (4.1%)	282 (5.8%)	0.615
Pipe	3 (<1%)	0	3 (<1%)	n/a
Oral	113 (2.1%)	3 (<1%)	110 (2.3%)	0.083
E‐cigarette	158 (3.0%)	15 (3.6%)	143 (2.9%)	0.595
Hookah	4 (<1%)	0	4 (<1%)	n/a
Bidis	0	0	0	n/a
Daily smoking	3950 (74.7%)	302 (72.1%)	3648 (75.0%)	0.212
Cigarettes per day	15.88 (11.29)	16.18 (11.85)	15.85 (11.24)	0.617
Time to smoke (dependence), min				
<5	2285 (43.2%)	183 (43.7%)	2102 (43.2%)	0.307
6–30	534 (10.1%)	43 (10.3%)	491 (10.1%)	
31–60	249 (4.7%)	18 (4.2%)	231 (4.7%)	
>60	440 (8.3%)	24 (5.7%)	416 (8.6%)	

Note

*N*s and percentages may not add up to 100 due to missing data. For the use of tobacco products, patients could endorse more than one response. *p‐*values indicate results from chi‐square tests or *t*‐tests of each variable.

Abbreviation: SD, standard deviation.

### 3.2 Inpatient interview outcomes

Results are shown in Table 2. Patients with and without cancer diagnoses were compared on importance in quitting and confidence in quitting. Patients with cancer diagnoses reported higher importance of quitting smoking (*M* = 3.92 [standard deviation, SD = 1.32]) than those without a cancer diagnosis (*M* = 3.77, SD = 1.36; *t* = 2.16, *p* = 0.031). However, patients with and without cancer diagnoses did not differ in confidence in quitting smoking (*t* = −0.31, *p* = 0.76). In unadjusted analyses, patients without cancer made significantly more quit attempts in the past year (*M* = 1.47, SD = 3.41) than those with a cancer diagnosis (*M* = 1.14, SD = 2.62; *t* = −2.36, *p* = 0.019). However, when controlling for age, this effect was no longer significant.

**TABLE 2:** Results of interview and follow‐up analyses

Variable (*M*, SD or *n*, %)	Patients with cancer diagnosis (*N* = 419)	Patients without cancer diagnosis (*n* = 4868)	*t* or *χ* ^2^, *p* value
Interview during admission			
Importance to quit (1–5)	3.92 (1.32)	3.77 (1.36)	2.16, 0.031*
Confidence to maintain quit (1–5)	3.50 (1.35)	3.52 (1.32)	−0.31, 0.76
Number of past year quit attempts	1.14 (2.62)	1.47 (3.41)	−2.36, 0.019*
Received medication while inpatient	246 (58.7%)	2992 (61.4%)	1.23, 0.267
Recommended discharge medication	183 (43.7%)	2573 (52.9%)	13.03, <0.001**
Discharge medication order complete	75 (17.9%)	649 (13.3%)	6.81, <0.01*
30‐Day follow‐up			
Smoking status (ITT)			
Quit	86 (20.5%)	502 (10.3%)	47.55, <0.001**
Ready to quit	41 (9.8%)	349 (7.2%)	
Not ready to quit	292 (69.7%)	4017 (82.5%)	
Completed call	200 (47.7%)	1773 (35.7%)	33.29, <0.001**
Smoking status (responders)			
Quit	120 (60%)	774 (43%)	17.84, <0.001**
Ready to quit	48 (24%)	516 (29.1%)	
Not ready to quit	32 (16%)	443 (25%)	

Note

Smoking status (responders) shows outcomes as a result of the ratio of patients that completed the telephone call. Smoking status (ITT) shows outcomes with an ITT approach, wherein non‐responders were coded as smoking and not ready to quit. Age‐adjusted analyses can be seen in the Table S1.

Abbreviations: ITT, intent‐to‐treat; SD, standard deviation.

* Indicates significance at *p* < 0.05.

** Indicates significance at *p* < 0.001.

When asked, 246/419 (58.7%) of patients with cancer diagnosis and 2992/4868 (61.5%) of patients without cancer diagnosis reported receiving medication in the hospital (mostly NRT); there was no significant difference between these groups (*χ* ^2^ = 1.23, *p* = 0.267). The TTP clinician recommended discharge medication for more patients without a previous cancer diagnosis (2573/4868, 52.9%) than cancer patients (183/419, 43.7%; *χ* ^2^ = 13.03, *p* < 0.001). However, a higher proportion of patients with cancer (75/419, 17.9%) were discharged with a completed medication order (signed off by the attending physician) compared to patients without cancer (649/4868, 13.3%; *χ* ^2^ = 6.81, *p* < 0.01).

### 3.3 Follow‐up outcomes

Follow‐up analyses are shown in Table 2, including adjusted sample sizes, ITT, and respondent‐only results. Analyzing within an ITT framework coded all patients who did not complete the 30‐day follow‐up phone call. Cancer patients self‐reported higher rates of abstinence (86/419, 20.5%) than those without a cancer diagnosis (502/4868, 10.3%; *χ* ^2^ = 45.75, *p* < 0.001). Patients without a cancer diagnosis showed a higher proportion disinterested in quitting (4017/4868 82.5%) than those with a previous cancer diagnosis (292/419, 69.7%). When examining respondents only, a higher percent of patients with cancer diagnoses completed the follow‐up survey than non‐cancer patients (*χ* ^2^ = 33.29, *p* < 0.001). Per self‐report one month following discharge, patients with cancer diagnoses differed from those without cancer in smoking status (*χ* ^2^ = 17.84, *p* < 0.001); a higher proportion of cancer patients (120/200, 60%) reported quitting than those without cancer (774/1773, 43%), and those without cancer reported a higher proportion of disinterest in quitting (443/1773 [25%] vs. 32/200 [16%]).

## 4 DISCUSSION

Results support the hypothesis that hospitalized patients with a cancer diagnosis reported higher rates of quitting smoking at follow‐up than patients without a cancer diagnosis. However, there were no significant differences in confidence in quitting following discharge. Interestingly, patients with a cancer diagnosis reported fewer past‐year quit attempts, although this effect was no longer significant when controlling for age. There were no significant differences between patients with and without cancer diagnoses on receiving smoking cessation medication in the hospital; however, patients with cancer were more likely to have pended medication suggestions completed by their physicians at discharge.

### 4.1 Cancer and smoking cessation

In general, documentation of tobacco use and cessation within the cancer patient population has yielded inconsistent results. A review from 2003 estimated that 46%–75% of patients were smoking at the time of diagnosis, with 14%–58% continuing to smoke after treatment.^25^ A more recently published study of intensive cessation support for patients demonstrated a 45% quit rate at 3 months.^26^ However, in another study of an opt‐out program where 730 cancer patients were automatically referred for phone‐based smoking cessation support, patients reported a 20% quitting rate.^27^ In the present study, patients with cancer history were more likely to have quit following the intervention; however, at the time of treatment, these patients declined services at the same rate as those without cancer history and also reported significantly fewer quit attempts in the past year. This suggests that, in general, patients with cancer may face similar challenges as those without cancer in deciding to quit smoking on their own. However, the effects of an intervention are somewhat stronger for those with a cancer history.

There are several possibilities with regard to why the study findings showed differences between patients with and without cancer diagnoses. First, a diagnosis of cancer may increase motivation for smoking cessation.^28^ Indeed, the initial diagnosis of cancer may lead to spontaneous, unassisted quit attempts for cancer patients,^29^ although this was not observed in the present sample based on the number of past‐year quit attempts. Though diagnosis of cancer might increase motivation to quit smoking, patient factors such as perceived severity of the disease, perceived benefits of engaging in health behavior change, perceived barriers, and low self‐efficacy for change can hinder smoking cessation efforts.^30^ Collectively, these factors may contribute to different success rates for quitting among patients within a spectrum of diagnoses and prognoses.^31^, ^32^, ^33^, ^34^ In the present study, recency of cancer diagnosis, treatment, or relatedness to hospital admission was not assessed. This allowed for a generalizable evaluation of cancer patients broadly. Future research could assess effects related to specifics of diagnosis, treatment, and temporal proximity on responses to interventions.

Second, hospitalized patients with a cancer history may have also been more receptive to and engaged in the intervention, and the results showing higher rates of follow‐up completion support this. Interestingly, these patients were also more likely to have their discharge medications completed by their physician, which suggests higher provider engagement in tobacco treatment. Patients who were hospitalized without a cancer diagnosis may have found it more challenging to cultivate personal significance within the intervention, and similarly, providers may not have prioritized tobacco treatment upon discharge.

The 60% self‐reported quit rate observed from the current study is higher than expected, particularly given the relatively low‐intensity support provided for cessation. Whereas these quit rates are encouraging, this could be in part be biased due to an expected 30% misreporting rate for tobacco use in cancer patients.^35^, ^36^, ^37^, ^38^ There is also a possibility that patients who quit smoking were more likely to answer follow‐up phone calls, and this is supported by the parallel high rate of quitting in patients without cancer (43%). Our ITT analysis (i.e., 20.5% vs. 10.3% abstinence) may be more accurate, while continuing to support the differences in cancer and non‐cancer patient responses to treatment.

### 4.2 Clinical implications

Although patients with cancer are motivated to quit smoking, they also experience unique barriers to quitting. The intervention discussed was more effective for patients with a cancer diagnosis than those without, which suggests that cancer patients may benefit from being approached multiple times about quitting smoking. That is, smoking cessation interventions should not be limited to encouraging quitting at the time of diagnosis or during the multitude of oncology appointments.^39^ Our study suggests that hospitalizations that occur after the diagnosis, whether related to or unrelated to cancer itself, represent an important opportunity for intervention that likely capitalize on previous motivations.

For those who continued smoking after a cancer diagnosis, receiving intervention from a TTP clinician at a separate, likely unrelated, appointment (hospital admission) could have also provided extra support needed to engage with the patients about a meaningful plan to change their smoking behavior. Indeed, there may have been variations in counseling content between patients in the present study based on health history disclosure and connection to smoking cessation motivation. Providers need not be discouraged regarding the aforementioned data showing concerning rates of smoking following diagnosis; rather, providers should be encouraged to continue “planting the seed” at each opportunity for tobacco treatment interventions, as they likely build upon one another.

In the present sample, cancer patients were more likely to be given medication upon discharge, which also could have contributed to higher success rates. In general, providers may face several barriers to completing discharge medication requests for smoking cessation, such as the lack of time or misconceptions about the medications requested. It appears that a history of cancer diagnosis may be associated with increased willingness and ability to complete these medication requests. This is, indeed, an area warranting further research and investigation, given the overall low rates of medication dispensing at discharge.

### 4.3 Limitations

Several limitations must be considered within the present analysis. The present sample may not represent the full population of hospitalized smokers due to the lack of ability to visit every patient, time constraints, and other challenges upon admission, such as hesitancy to disclose tobacco use to medical staff. Likewise, overall follow‐up rates were low and the reasons for these missing responses are unclear; thus, there are limitations to the ITT analyses used.^40^ Additionally, biochemical verification was not used to confirm abstinence at follow‐up.

Data collected during the TTP interviews are self‐report and were subjected to time constraints placed on clinicians, who were mindful of other providers needing time with patients. This led to some participants with only partial interview data. Although patients with cancer reported statistically higher ratings of importance to quit, the means of ratings between groups were similar. Thus, the effect size should be interpreted with caution. Further, follow‐up response rates were low. This is typical of such an intervention, and tobacco treatment programs are constantly considering ways to improve these systems. Nevertheless, the interpretation of the present analyses should be considered with missing data in mind. Finally, interview data should be interpreted with caution as the items used have not been psychometrically validated.

A low proportion of patients were identified as having a cancer diagnosis. Indeed, most cancer patients receive treatment outpatient and often do not require hospitalization, which limits the generalization of our results among all patients being treated for cancer. Similarly, other comorbid medical conditions not captured in the present study may have differed between groups. Future research could use more advanced propensity matching analyses to evaluate the extent of the effect of cancer diagnosis in the context of comprehensive medical histories. Further, the present sample of patients with cancer diagnoses may have, prior to hospitalization, been exposed to smoking cessation telephone counseling through the cancer hospital when being treated as an outpatient. However, these data are not available through patients’ hospitalization records; therefore, we are unable to determine to what extent our cancer diagnosis sample received prior treatment. There was no overlap in treatments at the time of inpatient hospitalization. Along similar lines, past exposure to other smoking cessation counseling and treatment was not captured.

## 5 CONCLUSION

The results of the present study suggest that patients with cancer diagnoses benefit from an intervention delivered during an inpatient hospitalization. Future interventions should capitalize upon this effect to tailor brief interventions for cancer patients, as well as to increase the salience of such interventions for the non‐cancer populations. In addition, future research needs to work toward optimizing motivation for smoking cessation while admitted to a hospital and on improving quit rates for all admitted patients, regardless of diagnosis.

## ETHICS STATEMENT

This study was exempt from participant consent and approved by the Medical University of South Carolina (MUSC) Institutional Review Board (IRB), which adheres to the U.S. Federal Policy for the Protection of Human Subjects.

## DATA AVAILABILITY STATEMENT

Research data are originated from electronic health records and are not shared due to privacy restrictions.

## CONFLICTS OF INTEREST

B.A.T. and K.M.C. are consultants to Pfizer on an Advisory Board on e‐cigarettes and ways to improve smoking cessation delivery in health care settings. B.A.T. and K.M.C. testify on behalf of plaintiffs who have filed litigation against the tobacco industry.

## Supporting information


**TABLE** **S1**. Results of interview and follow‐up analyses, adjusted for age

## References

[1] National Center for Chronic Disease Prevention and Health Promotion (US) Office on Smoking and Health . The health consequences of smoking—50 years of progress: A report of the surgeon general.Atlanta,GA: Centers for Disease Control and Prevention (US), 2014. PMID: 24455788. 24455788

[2] Substance Abuse and Mental Health Services Administration, Office of the General Surgeon . Smoking Cessation: A Report of the Surgeon General. US Department of Health and Human Services; 2020.

[3] Warren GW , Simmons VN . Tobacco use and the cancer patient. In: Lawrence TL , ed. DeVita, Hellman, and Rosenberg's Cancer: Principles and Practice of Oncology. 11th ed. Lippincott, Williams, & Wilkins; 2019.

[4] Warren GW , Alberg AJ , Cummings KM , Dresler C . Smoking cessation after a cancer diagnosis is associated with improved survival. J Thorac Oncol. 2020;15(5):705‐708. 32197939 10.1016/j.jtho.2020.02.002

[5] Shields PG , Herbst RS , Arenberg D , et al. Smoking cessation, version 1.2016, NCCN clinical practice guidelines. J Natl Compr Canc Netw. 2016;14(11):1430‐1468. 27799513 10.6004/jnccn.2016.0152

[6] Orleans CT , Kristeller JL , Gritz ER . Helping hospitalized smokers quit: new directions for treatment and research. J Consult Clin Psychol. 1993;61(5):778‐789. 8245275 10.1037//0022-006x.61.5.778

[7] Freund M , Campbell E , Paul C , et al. Smoking care provision in hospitals: a review of prevalence. Nicotine Tob Res. 2008;10(5):757‐774. 18569750 10.1080/14622200802027131

[8] Hollis JF , Bills R , Whitlock E , Stevens VJ , Mullooly J , Lichtenstein E . Implementing tobacco interventions in the real world of managed care. Tob Control. 2000;9(suppl 1):i18‐i24. 10688926 10.1136/tc.9.suppl_1.i18 PMC1766258

[9] Wolfenden L , Campbell E , Walsh RA , Wiggers J . Smoking cessation interventions for in‐patients: a selective review with recommendations for hospital‐based health professionals. Drug Alcohol Rev. 2003;22(4):437‐452. 14660134 10.1080/09595230310001613967

[10] Rojewski AM , Bailey SR , Bernstein SL , et al. Considering systemic barriers to treating tobacco use in clinical settings in the United States. Nicotine Tob Res. 2019;21(11):1453‐1461. 29917118 10.1093/ntr/nty123 PMC6941704

[11] Taylor CB , Miller NH , Cameron RP , Fagans EW , Das S . Dissemination of an effective inpatient tobacco use cessation program. Nicotine Tob Res. 2005;7(1):129‐137. 15804685 10.1080/14622200412331328420

[12] Cartmell KB , Dismuke CE , Dooley M , et al. Effect of an evidence‐based inpatient tobacco dependence treatment service on 1‐year postdischarge health care costs. Med Care. 2018;56(10):883‐889. 30130271 10.1097/MLR.0000000000000979 PMC6136961

[13] Richter KP , Ellerbeck EF . It's time to change the default for tobacco treatment. Addiction. 2015;110(3):381‐386. 25323093 10.1111/add.12734

[14] Rigotti NA , Munafo MR , Stead LF . Smoking cessation interventions for hospitalized smokers: a systematic review. Arch Intern Med. 2008;168(18):1950‐1960. 18852395 10.1001/archinte.168.18.1950 PMC4500120

[15] Cartmell KB , Dooley M , Mueller M , et al. Effect of an evidence‐based inpatient tobacco dependence treatment service on 30, 90 and 180‐day hospital readmission rates. Med Care. 2018;56(4):358‐363. 29401186 10.1097/MLR.0000000000000884 PMC5851827

[16] McBride CM , Emmons KM , Lipkus IM . Understanding the potential of teachable moments: the case of smoking cessation. Health Educ Res. 2003;18(2):156‐170. 12729175 10.1093/her/18.2.156

[17] Gritz ER , Fingeret MC , Vidrine DJ , Lazev AB , Mehta NV , Reece GP . Successes and failures of the teachable moment. Cancer. 2006;106(1):17‐27. 16311986 10.1002/cncr.21598

[18] Ferrucci LM , Cartmel B , Turkman YE , et al. Causal attribution among cancer survivors of the 10 most common cancers. J Psychosoc Oncol. 2011;29(2):121‐140. 21391066 10.1080/07347332.2010.548445 PMC3074193

[19] Warren GW , Dibaj S , Hutson A , Cummings KM , Dresler C , Marshall JR . Identifying targeted strategies to improve smoking cessation support for cancer patients. J Thorac Oncol. 2015;10(11):1532‐1537. 26317914 10.1097/JTO.0000000000000659

[20] Nahhas GJ , Cummings KM , Talbot V , Carpenter MJ , Toll BA , Warren GW . Who opted out of an opt‐out smoking‐cessation programme for hospitalised patients? J Smok Cessat. 2016;12(4):199‐204.

[21] Nahhas GJ , Wilson D , Talbot V , et al. Feasibility of implementing a hospital‐based "opt‐out" tobacco‐cessation service. Nicotine Tob Res. 2017;19(8):937‐943. 27928052 10.1093/ntr/ntw312 PMC10615132

[22] Warren GW , Marshall JR , Cummings KM , et al. Automated tobacco assessment and cessation support for cancer patients. Cancer. 2014;120(4):562‐569. 24496870 10.1002/cncr.28440 PMC4482255

[23] Transdisciplinary Tobacco Use Research Center (TTURC) Tobacco Dependence , Baker TB , Piper ME , et al. Time to first cigarette in the morning as an index of ability to quit smoking: implications for nicotine dependence. Nicotine Tob Res. 2007;9(suppl 4):S555‐S570. 18067032 10.1080/14622200701673480 PMC2933747

[24] Ellenberg JH . Intent‐to‐treat analysis versus as‐treated analysis. Drug Inform J. 1996;30(2):535‐544.

[25] Cox LS , Africano NL , Tercyak KP , Taylor KL . Nicotine dependence treatment for patients with cancer. Cancer. 2003;98(3):632‐644. 12879483 10.1002/cncr.11538

[26] Cinciripini PM , Karam‐Hage M , Kypriotakis G , et al. Association of a comprehensive smoking cessation program with smoking abstinence among patients with cancer. JAMA Netw Open. 2019;2(9):e1912251. 10.1001/jamanetworkopen.2019.12251 31560387 PMC6777393

[27] Amato KA , Reid ME , Ochs‐Balcom HM , et al. Evaluation of a dedicated tobacco cessation support service for thoracic cancer center patients. J Public Health Manag Pract. 2018;24(5):E12‐E19. 29278577 10.1097/PHH.0000000000000674 PMC6014867

[28] McBride CM , Ostroff JS . Teachable moments for promoting smoking cessation: the context of cancer care and survivorship. Cancer Control. 2003;10(4):325‐333. 12915811 10.1177/107327480301000407

[29] Campling BG , Collins BN , Algazy KM , Schnoll RA , Lam M . Spontaneous smoking cessation before lung cancer diagnosis. J Thorac Oncol. 2011;6(3):517‐524. 21258255 10.1097/JTO.0b013e318208c7da

[30] Champion VL , Skinner CS . The health belief model. In: Glanz K, Rimer BK, Viswanath K, ,eds. Health Behavior and Health Education: Theory, Research, and Practice. 4th edn. (pp. 45‐65). Jossey‐Bass; 2008.

[31] Schnoll RA , Martinez E , Langer C , Miyamoto C , Leone F . Predictors of smoking cessation among cancer patients enrolled in a smoking cessation program. Acta Oncol. 2011;50(5):678‐684. 21534846 10.3109/0284186X.2011.572915

[32] Martínez Ú , Brandon TH , Sutton SK , Simmons VN . Associations between the smoking‐relatedness of a cancer type, cessation attitudes and beliefs, and future abstinence among recent quitters. Psycho‐Oncology. 2018;27(9):2104‐2110. 29785718 10.1002/pon.4774 PMC6156937

[33] Bryant J , Boyes AW , Hall A , Girgis A , D'Este C , Sitas F . Prevalence and factors related to smoking and smoking cessation 6 months following a cancer diagnosis: a population‐based study. J Cancer Surviv 2016;10(4):645‐653. 26758587 10.1007/s11764-015-0510-7

[34] Sosnowski R , Kamecki H , Bjurlin MA , Przewoźniak K . The diagnosis of bladder cancer: are we missing a teachable moment for smoking cessation? Transl Androl Urol. 2019;8(suppl 3):S318‐S321. 31392157 10.21037/tau.2019.05.15 PMC6642978

[35] Morales NA , Romano MA , Michael Cummings K , et al. Accuracy of self‐reported tobacco use in newly diagnosed cancer patients. Cancer Causes Control. 2013;24(6):1223‐1230. 23553611 10.1007/s10552-013-0202-4 PMC4477518

[36] Warren GW , Arnold SM , Valentino JP , et al. Accuracy of self‐reported tobacco assessments in a head and neck cancer treatment population. Radiother Oncol. 2012;103(1):45‐48. 22119370 10.1016/j.radonc.2011.11.003 PMC3327779

[37] Marin VP , Pytynia KB , Langstein HN , Dahlstrom KR , Wei Q , Sturgis EM . Serum cotinine concentration and wound complications in head and neck reconstruction. Plast Reconstr Surg. 2008;121(2):451‐457. 18300961 10.1097/01.prs.0000297833.53794.27

[38] Khuri FR , Kim ES , Lee JJ , et al. The impact of smoking status, disease stage, and index tumor site on second primary tumor incidence and tumor recurrence in the head and neck retinoid chemoprevention trial. Cancer Epidemiol Biomarkers Prev. 2001;10(8):823‐829. 11489748

[39] Toll BA , Brandon TH , Gritz ER , Warren GW , Herbst RS . Assessing tobacco use by cancer patients and facilitating cessation: an American Association for Cancer Research policy statement. Clin Cancer Res. 2013;19(8):1941‐1948. 23570694 10.1158/1078-0432.CCR-13-0666 PMC5992896

[40] Gupta SK . Intention‐to‐treat concept: a review. Perspect Clin Res. 2011;2(3):109‐112. 21897887 10.4103/2229-3485.83221 PMC3159210

