# Supplementary material for: Associations between cancer diagnosis and patients’ responses to an inpatient tobacco treatment intervention
Source: Cancer Med. 2021 Jul 1;10(15):5329–37. doi: 10.1002/cam4.4082 (PMC8335828; doi:10.1002/cam4.4082)
Supplement: Supplementary file 1 — TABLE S1. Results of interview and follow‐up analyses, adjusted for age [file CAM4-10-5329-s001.docx]

Supporting information. *Results of interview and follow-up analyses, adjusted for age*

| **Variable (M, SD or n, %)** | **F or B  (cancer diagnosis)** | ***p* value** |
| --- | --- | --- |
| Importance to quit (1-5) | 4.73 | .03 |
| Confidence to maintain quit (1-5) | .071 | .79 |
| Number of past year quit attempts | 3.404 | .06 |
| Received medication while inpatient | 1.11 | .30 |
| Recommended discharge medication | .712 | .01 |
| Discharge medication order complete | 1.43 | <.001 |
| ***30-day follow-up*** |  |  |
| Smoking status (ITT) ^a^  Quit  Ready to quit  Not ready to quit | Ref  2.15  1.66 | -  <.001  .005 |
| Completed call | 1.65 | <.001 |
|  |  |  |
| Smoking status (responders) ^a^  Quit  Ready to quit  Not ready to quit | Ref  1.35  1.41 | -  .002  .032 |

Note: To adjust for age, analyses were run as ANCOVA, binary logistic regression, or multinomial logistic regression. ^a^ Odds presented are for those without a cancer diagnosis (reference category = cancer history). Smoking status (responders) shows outcomes as a result of the ratio of patients that completed the telephone call. Smoking status (ITT) shows outcomes with an intent-to-treat approach, wherein non-responders were coded as smoking and not ready to quit.
